# Mothering from the Margins: Ethnographic Reflections on the Gendered Politics of Rohingya Mothers in India

**DOI:** 10.1177/01979183251331872

**Published:** 2025-04-22

**Authors:** Raksha Gopal

**Affiliations:** Geneva Graduate Institute, Switzerland

**Keywords:** forced displacement, migration governance, gender

## Abstract

This article analyses the everyday experiences of stateless Rohingya refugee women mothering and raising families within refugee settlements in Delhi. Centering the narratives of refugee women, I argue that motherhood is a site for the governance of migration, where insecurities are felt and agency may be expressed. First, I illustrate the tensions between the gendered expectations on Rohingya mothers — rooted in intersecting social, cultural and familial norms — and the insecurities they face as a result of their displacement in India. These gendered expectations increase women's caregiving and social reproduction responsibilities while simultaneously limiting their mobility, access to employment, resources, and social support. Secondly, I demonstrate how Rohingya mothers continuously negotiate a sense of security for themselves and their children against the insecurities engendered by formal governance systems of the Indian State. Finally, I explore how non-state humanitarian organizations and NGOs play a crucial role in the governance of refugee women, shaping not only their access to resources and aid but also women's identities and roles as mothers. As a result, Rohingya women become key actors linking state and non-state governance to the everyday practices of motherhood.

## Introduction

Jannat^1^ and I are sitting across from each other, cross-legged and shivering against the cold floor in her *jhuggi*^2^ in the middle of a slum in southeast Delhi. She — a Rohingya woman in her late teens — gently cradles her young daughter on her lap as she ruminates about her family's life in the settlement. Jannat was born in Myanmar and raised as a refugee in Bangladesh after fleeing the violent persecution of her community by the Burmese military and armed groups in Rakhine State. She was then married at fifteen years of age and brought to India as the bride of another Rohingya man from her community. In the last four years, she has given birth to three stateless babies in a government hospital in New Delhi, but only one of them has survived. Jannat has multiple theories about what happened, ranging from the more extreme (evil *djinns*) to those that evoke superstitions (the evil eye). However, the more likely cause is that buying food, even a few pints of milk, essential for proper nutrition during pregnancies, costs more than her husband's monthly salary, especially as humanitarian aid has declined. As an undocumented refugee in India, her husband is unable to seek employment. So, her family struggles to make ends meet. She explains that her life has changed drastically since becoming a mother in a community where gender roles are divided strictly between men and women:

I have always been a refugee since I was old enough to make sense of the world. So, it does not bother me. However, since I have become a mother, my responsibilities have increased […] you have to take care of children, cook for them, keep them safe, and when I look at the Indian children with their mothers, in their big houses, I wish for a bigger house and a better life for my children. But I think my wish will always remain a dream…until we get our own country. (excerpt from interview, November 23, 2023)

Jannat's story echoes those of several Rohingya refugee women whom I met during my fieldwork in Delhi between 2023 and 2024, highlighting the challenges of mothering while displaced and stateless. Her narrative alludes to the tensions between the everyday material, socio-economic, and legal insecurities Rohingya women experience, the gendered expectations on them to perform care and social reproduction, and their everyday aspirations for an impossible belonging in destination countries like India.

My conversations with Jannat occurred against the backdrop of increasing insecurities among Rohingya refugees in India (Mitra and Şahin-Mencütek 2024). Labeled as “illegal,” they remain in constant fear of detention or deportation because the Indian Government does not recognize the refugee cards issued by the Office of the United Nations High Commissioner for Refugees (UNHCR) (Tiwari, Johar and Field 2024). In this article, I address two questions. Firstly, how do Rohingya refugee mothers negotiate the everyday gendered insecurities they face in India? Secondly, what does a focus on motherhood reveal about the intersection between gender, displacement, and the transnational governance of refugees?

The insecurities faced by the Rohingya in India are rooted in histories of violent systemic exclusion and racialization in Myanmar (Farzana 2017). Statelessness, restricted access to civil and political rights, land dispossession, and gendered expectations have long shaped Rohingya women's everyday lives (Frydenlund 2020). Following waves of ethnic persecution and gender-based violence, particularly between 2012 and 2017, several Rohingya women migrated with their families to neighboring countries like India in search of security, better livelihoods, and resettlement in third countries. Some were also brought to India as brides in arranged marriages to Rohingya men or were victims of trafficking. In this context, many Rohingya women are giving birth and raising their families in destination countries, where they face restricted access to essential infrastructure, such as housing, employment, education, and reproductive healthcare, while being subjected to surveillance, detention, and humanitarian interventions. Despite these challenges, women like Jannat are expected to be ‘good mothers' and caregivers within their communities, further entrenching gendered hierarchies. Thus, Rohingya mothers continuously negotiate these layered insecurities in their everyday lives to survive. Crucially, these insecurities are gendered, as Rohingya women often face greater burdens of care and social reproduction even as they face other socioeconomic and legal insecurities (Field, Pandit and Rajdev 2023).

In this article, I explore these gendered insecurities further by focusing on the mothering practices performed by Rohingya women as they navigate everyday life in Delhi. Informed by feminist International Relations (IR), I recognize the ‘everyday' as a site permeated by gendered relations of power that shape women's experiences significantly, (Blomqvist, Olivius and Hedström 2021) and where “insecurities are felt, feared and negotiated” (Hedström 2021, 371). This paper acknowledges the gendered nature of power and governance in global politics (Enloe 2014; Prügl and Ann Tickner 2018). This allows us to understand how the various practices and performances of motherhood, often deemed apolitical, are constitutive of the different dimensions of political life (Ruddick 1989; Robinson and Confortini 2014; Hall, Weissman and Shepherd 2020). Central to motherhood are the embodied and affective labors necessary for the production of life — or social reproduction (Elias and Rai 2019) — including making babies, parenting, caregiving, procuring food, clothing, shelter, education, and healthcare.

Based on my findings, I make three arguments. Firstly, I illustrate the tensions between the gendered expectations on Rohingya mothers — rooted in intersecting social, cultural and familial norms — and the insecurities they face as a result of their displacement in India. These gendered expectations increase women's caregiving and social reproduction responsibilities, while simultaneously limiting their mobility, access to employment, resources, and social support in displacement, thus intensifying their insecurities. Secondly, I demonstrate how Rohingya mothers continuously negotiate a sense of security for themselves and their children against the insecurities engendered by formal governance systems of the Indian State. Finally, I explore how non-state humanitarian organizations play a crucial role in the governance of Rohingya refugee women, shaping not only their access to resources and aid but also their identities and roles as mothers. These organizations mobilize women's identities as mothers, for instance by recruiting them as volunteers for their developmental interventions and programmes and extending their caregiving practices to the broader community. As a result, Rohingya women become key actors linking state and non-state governance to the everyday practices of motherhood.

This paper integrates feminist migration research and feminist IR literature, highlighting that motherhood is a crucial site for the governance of refugee women, where insecurities are experienced and agency may be expressed. It contributes to feminist migration research, which recognizes refugee women's agency and the significance of their acts of care and social reproduction for the survival of their communities (Constable 2021; Rottmann and Nimer 2021; Al-Dabbagh 2022; Lamp-Miechowiecki 2024). While the literature on conflict and peacebuilding in Myanmar has examined care and social reproduction among other displaced Burmese communities (Pearson and Kusakabe 2012; Hedström et al. 2023; Lingham and Johnston 2025), there is limited scholarship on Rohingya women beyond portrayals of victimhood and gender-based violence, especially in conditions of prolonged displacement. Instead, focusing on Rohingya women's everyday lives as mothers and homemakers can reveal the complex and multi-dimensional aspects of their identities and their resilience and resistance to violent marginalization (Frydenlund 2020). In this vein, I argue that motherhood, a crucial part of many women's lives, offers a critical lens to analyze the experiences of forcibly displaced communities like the Rohingya in India. Thus, this paper foregrounds narratives often obscured by masculinized and securitized discourses on the Rohingyas in India and globally (Malaviya 2020).

## Motherhood as a Critical Lens of Analysis for Migration

This paper draws on feminist IR, which conceptualizes motherhood as a gendered site of power that is diversely constructed and practiced across different settings and entwined with dimensions of political life, such as race, ethnicity, class, sexuality, nationality, and religion (Ruddick 1989; Hall, Weissman and Shepherd 2020). Foregrounding motherhood allows us to analyze how refugee women's everyday lives are regulated through their identities and responsibilities as mothers. It also reveals how their gendered labor is incorporated into broader frameworks of refugee governance, often heightening their existing insecurities. Far from being apolitical, motherhood can be a space for negotiating gender roles and resistance (Sjoberg and Gentry 2007).

Simultaneously, feminist migration research emphasizes that gendered relations of power and difference intersect with other identity categories fostering exclusions that shape displaced women's lived experiences (Freedman 2012; Bélanger and Silvey 2020). By restricting access to essential state services, such as employment, education, housing, and healthcare, destination states often fail to support refugees’ welfare and caring responsibilities. At times, they directly obstruct them through policies, legislation, or practices that undermine refugees’ rights, thereby producing multiple insecurities (Valiavicharska 2020). Thus, refugee women experience heightened exposure to gender-based violence (GBV), restricted mobility, and reduced access to education and reproductive healthcare (Freedman 2016).

The lack of access to essential resources amplifies demands for care and social reproduction, disproportionately affecting women, who often bear the primary responsibility for caregiving (Kofman and Raghuram 2018). Feminist political economists have long emphasized the centrality of the violent exploitation and expropriation of women's unwaged care and social reproduction labor to the gendered and racialized structures of modern nation-states and their economies (Fraser 2016; Bhattacharya 2017; Federici 2020). Additionally, this labor is essential for sustaining the workforce and ensuring the everyday survival of communities, especially during periods of crisis. Despite being restricted to the so-called “private” spheres of the home, the performance of these labors extends into other aspects of social and political life. At times, migrant women contribute to their households financially, especially in contexts where securitization and gendered migration policies affect men's mobilities and restrict their employment opportunities (Akhter and Kusakabe 2014). This reshapes traditional gender roles and forces women to bear a double burden of performing unpaid and undervalued domestic tasks and waged labor, while enduring the trauma of conflict and migration (Lingham and Johnston 2025).

These insecurities are reinforced by state and non-state actors involved in refugee governance frameworks, which are inherently gendered. Through immigration policies, discourses, development interventions, and aid delivery, these actors shape refugee women's experiences by politicizing motherhood and reproduction (Whiteford and Eden 2011). State actors often portray refugee women as both threatening and vulnerable to justify restrictive immigration policies that seek to surveil, police and control reproductive choices, restrict asylum, and expose women to arrests, deportations, and family separations (Tyler 2013).

Previous scholarship also highlights that humanitarian organizations engage in gendered governance, reinforcing refugee women's vulnerability by portraying them as passive victims to attract support for their interventions (Johnson 2011). Saltsman (2023) argues that this is a form of gendered violence that intensifies refugee women's insecurities and gender hierarchies within communities. Through developmental interventions, they reproduce specific gendered expectations and norms around motherhood to govern women's reproductive bodies, and, at times, devalue indigenous practices and norms (Olivius 2015; Clark 2016).

While feminists critique the undervaluation of women's everyday labors of care and social reproduction and emphasize the violence inherent in them, they also recognize it as a site for agency. (Elias and Rai 2019). Feminist migration research moves beyond framing refugee women as embodiments of “bare life” (Agamben 1998). Instead, it highlights how refugee women negotiate agency even in conditions of extreme insecurity during displacement, especially within the “private” spheres of everyday life, such as motherhood (Kanal and Rottmann 2021). Research also underscores the significance of mothering practices for creating a home in displacement and fostering belongingness among refugee children against exclusionary politics in destination countries (Al-Dabbagh 2022). In some cases, refugee women actively leverage their identities as mothers to contest state violence and demand recognition of their rights through protests and collective mobilization (Fujiwara 2008). However, refugee mothers also engage in subtle forms of negotiation and resistance every day to ensure the well-being of their families. For example, refugee mothers perform a wide range of visible (e.g., working without legal authorization, negotiating with state and non-state actors for resources) and invisible (e.g., avoiding the police, trying to stay abreast of the news) practices to protect their children from state securitization (Constable 2021). In this way, motherhood is entangled with issues of governance and security.

This paper adopts motherhood as a critical lens to examine the everyday experiences and practices of Rohingya refugee mothers. It analyzes how gendered insecurities are produced and reproduced by various state, non-state, community and individual actors within refugee camps and settlements, and how Rohingya women negotiate them. In doing so, I respond to calls to reject the analytical centrality of the state in migration research by recognizing migration as a site of negotiation “where people on the move play a constitutive role” (Squire 2020).

## Context

The Rohingya, a predominantly Muslim minority community from Rakhine State, Myanmar, have faced decades of systemic exclusion, land dispossession, racialized discrimination, statelessness, and waves of persecution in the region (Farzana 2017). Millions have fled to neighboring countries in South and Southeast Asia, particularly Bangladesh, where securitization practices further marginalize them (Chaudhury and Samaddar 2018).

Rohingya women's everyday experiences are shaped by pre-existing gender hierarchies and community norms that confine them to the private spaces of the home, limiting their visibility in public life (Hutchinson 2018; Digidiki and Bhabha 2021). The practice of *purdah*, including the use of the headscarves and *burka* or the veil, reinforces these socially constructed hierarchies (Frydenlund 2020). Rohingya girls are often forced to stay at home after reaching reproductive age for reasons of security and honor. These practices expose the Rohingya to racialized rhetoric of their backwardness (Frydenlund 2020).

In Myanmar and Cox's Bazar in Bangladesh, rampant GBV due to conflict and displacement has worsened gendered restrictions on Rohingya women (Hutchinson 2018). Histories of GBV, domestic violence, child marriages, and teenage pregnancies restrict women's access to education and employment (Jose 2022). These forms of physical and structural violence are frequently associated with shame and guilt, preventing women from raising concerns publicly (Akhter and Kusakabe 2014; Jose 2022).

Crucially, motherhood is central to women's experiences of insecurity in displacement. For Rohingya women, motherhood is rarely a choice. When I naively asked a Rohingya woman in Delhi if she had always wanted to be a mother, my interpreter, Z, herself a Rohingya, was unable to translate the question. Instead, she asked, “*Does that really happen?*” — revealing the gendered expectation for women to be reproducers and caregivers within the community. In this vein, Frydenlund (2020) argues that Rohingya women's unwaged domestic and emotional labor are important because they subsidize the low wages earned by male members of their families in informal labor markets and ensures the continued survival of their communities under racialized oppression. While focusing on Rohingya motherhood can reveal the layered insecurities of women within the community, it can also unravel the complex ways in which they must continuously negotiate a sense of security and belonging, especially as many children are born into prolonged statelessness.

Research on Myanmar's militarization and economic reforms indicates that the privatization, liberalization, and informalization of labor have subjected the lower classes, especially women, to increasing precarity, compelling many to migrate or engage in exploitative labor regimes (Campbell 2022). While the situation of the Rohingya highlights similar trends of precarization and exploitation, it is further exacerbated by their lack of citizenship, which excludes them from the state entirely. Beyond economic insecurities, they are subjected to racialized othering and are criminalized for simply existing. In this context, Rohingya women's gendered labor is not only integral to informal economies at the margins of nation-states but also to the intergenerational survival of a persecuted stateless community. Rodriguez and Griffiths (2022) assert that in contexts where the mothering body is unwanted, even the act of continuing to mother can be understood as a form of resistance to insecurity.

Building on the broader context of Rohingya women's gendered experiences, the urban setting in Delhi provides a unique lens to understand the intersection of motherhood, gendered insecurities, and the governance of refugees. Over 22,000 Rohingya refugees have sought asylum in India since 2017, with likely higher numbers including those who arrived during earlier periods of persecution (Sullivan and Sur 2023). Rohingya refugees are scattered across Indian cities, including Delhi, residing in informal settlements, where they face challenges related to their legal status and access to resources. India's fragmented approach to the governance of refugees means that the Rohingya policy has been prone to drastic changes and politicization, including a brief period of regularization of Long-Term Visas to the most recent crack-down on the refugees through mass detentions, threats of deportations, and strict mobility restrictions (Barthwal-Datta and Singh 2024; Mitra 2025).

**Figure 1.**
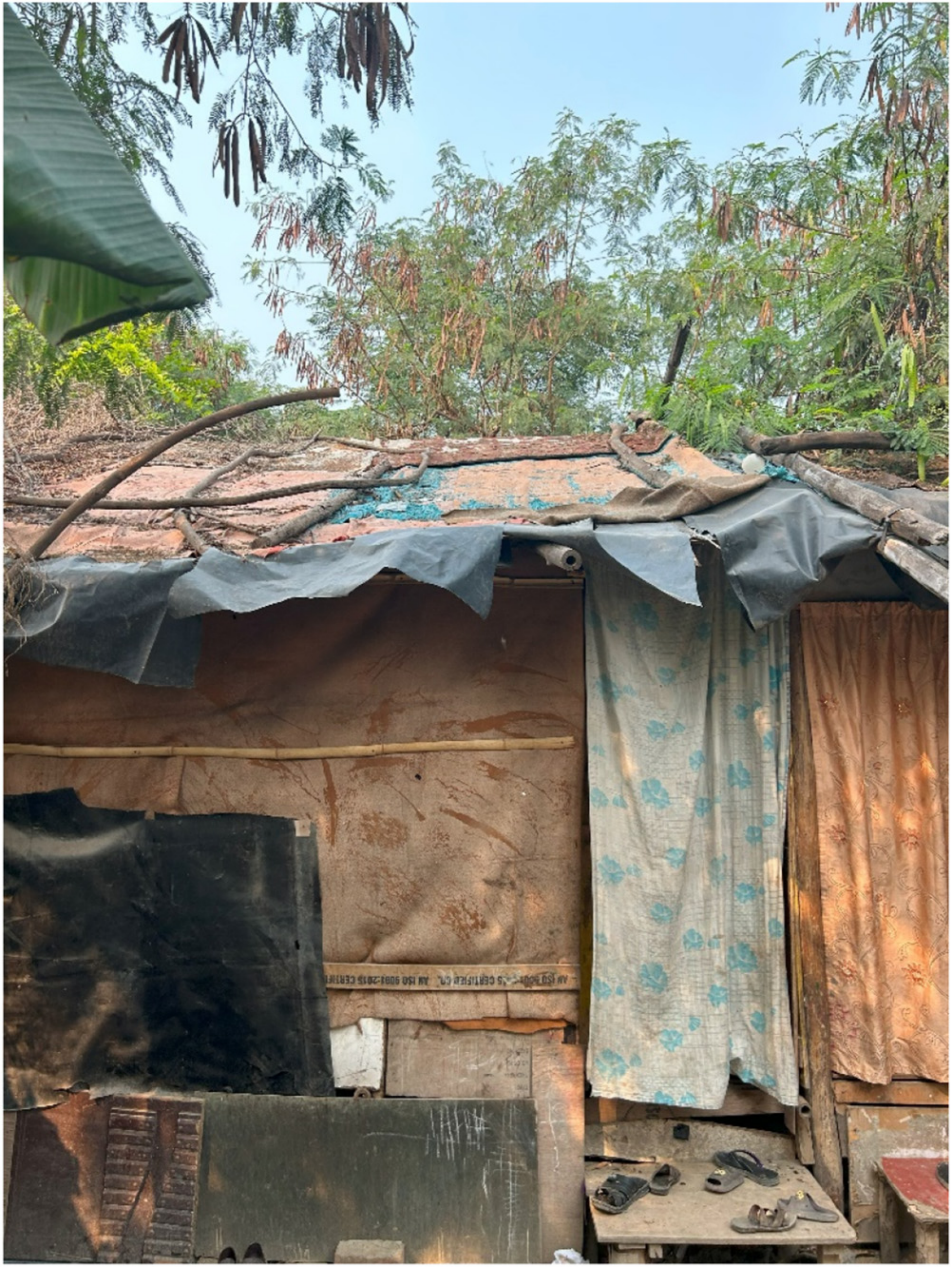
*Jhuggi* of a Rohingya family in Delhi.

In Delhi, Rohingya refugees live in precarious housing arrangements, such as rented rooms or slum dwellings called “*jhuggis”* (Figure 1), that are prone to fires, floods, and decay. Their “illegal” settlement has been a major electoral issue in Delhi, heightening insecurities among the Rohingya with many of them fearing eviction from these settlements (Apoorvanand 2022). Rohingya migration to India is also occurring alongside the broader discourse on citizenship, right-wing nationalism, and migration laws in the country. Migration to Delhi among Rohingya women is often driven by family reunification, arranged marriage proposals, job opportunities, hopes of resettlement, and cross-border trafficking (Kaveri 2020; Abbas and Hemadri 2022). Within settlements, gender roles are strictly divided. While men leave home early in the morning in search of daily wage labor, it is usually the women who stay behind cooking, cleaning, collecting water, and tending to children, thus bearing a significant burden of care labor and social reproduction.

## Methods

This paper draws on seven months of ethnographic research between 2023 and 2024 in several Rohingya settlements located in Delhi, focusing on areas with higher concentrations of Rohingya refugee families.^3^ In these settlements, refugees reported severe difficulties with access to electricity, potable water, evictions, and unaffordable rents. My findings are based on participant observation during daily visits to the homes of Rohingya refugees, NGO centers, and community meetings, 53 semi-structured interviews (47 women, 6 men), and informal encounters I had with refugee families, NGO representatives, journalists, and other academics. Rohingya mothers interviewed were between 19 and 50 years of age, with an average of four children under the age of 12.^4^ Most of them had given birth in India, either in government hospitals or at home where they were assisted by midwives. Five of the women interviewed were single mothers, separated from their husbands by death, detention, or undisclosed reasons. Many women lacked formal education and were illiterate but had received some form of religious schooling. Most of them stayed at home, while a few worked as seamstresses or engaged in other informal jobs (e.g., janitorial work, rag-picking, vending). The men interviewed were between 25 and 80 years of age and were usually involved in informal work as daily wage laborers in construction, factories, or vending. Participants had been residing in Delhi for varying lengths of time, with the majority arriving between 2011 and 2012. Participants were selected using personal connections and snowball sampling. All interviews were conducted in Hindi, and occasionally in Rohingya, and were later translated into English. Conversations centered on routines, access to essential services, mobility, experiences with state and non-state actors, relationships with children and family members, cultural practices, and aspirations for resettlement.

Aligning with feminist methodologies, I recognize the value of situating, locating, and positioning knowledge within the specific social and cultural contexts of both the researcher and the researched (Haraway 1988; Vrasti 2008). During my time in the field, I embodied various shifting positionalities that became integral parts of my visible and invisible ethnographic toolkit (Reyes 2020). At times, my identity as a young woman facilitated conversations with women on the intimate aspects of their everyday lives. However, as an Indian national from Delhi currently studying at a Western institution, I also had to navigate the intersectional power asymmetries, which were evident and unavoidable. Although I had access to and knowledge about the field sites, disparities in language, literacy, financial precarity, and crucially, the statelessness of my refugee participants created clear hierarchies of privilege, revealing the extractive nature of ethnographic research (Fujii 2012; Knott 2019).

Therefore, critically reflecting on positionality during and after fieldwork has been a central part of my research process. To decolonize the research, I prioritized treating refugee participants as experts in their experiences (Taha 2022). This involved acquiring informed verbal consent, seeking feedback on research topics, and engaging in a collaborative process of meaning-making. Interviews necessitated ongoing translation and negotiation of Rohingya and Hindi terms to better reflect the narratives of Rohingya women. Moreover, given the risks of detention and surveillance, I met participants in their homes or at NGO centers mindful of women's restricted mobility outside settlements. Although I met and conversed with most participants individually, researching the every day required acknowledging the fluidity of such interactions. Interviews were occasionally interrupted by neighbors and family members, resulting in group conversations where women's narratives and opinions were shaped in dialogue. The semi-structured format allowed participants to share freely, respecting their privacy. I also adapted my methods to align with women's responsibilities and avoid disrupting their routines.

To build trust, I gradually introduced myself to the community by visiting and volunteering at NGO centers. Later, I collaborated with a community member who assisted with interpretations, facilitated interviews and provided occasional translations, and who was remunerated for their time. Hiring interpreters in ethnographic research, however, is not without its challenges, as it carries the potential for exploitation and extraction (Abedi-Dunia, Toppo and Vincent 2023). Acknowledging these concerns, I worked closely with the interpreter, whose regular feedback and advice enriched the research. This collaboration facilitated the co-production of knowledge (Squire 2020), blending the the interpreter's insights, my participants’ perspectives, and my own reflections throughout this research.

## Findings

In the following sections, I explore the everyday practices of Rohingya mothers performed in the context of gendered expectations intensified by their displacement and statelessness in Delhi. These practices contribute to gendered insecurities that are imposed at multiple levels by the community, state, and non-state actors. However, they are also a key avenue through which Rohingya women negotiate a sense of security and belonging for themselves and their families. This contributes to an understanding of how Rohingya refugee women are often governed through their identities and roles as mothers.

### Gendered Expectations and Insecurities Around Motherhood

“What is the life of a woman? It is *this* only”, Aarifa says from the stoop of her house, flailing her arms towards her children and the jhuggi behind her. There is a wide smirk on her face as she turns to Z (my interpreter) and says something hurriedly. Z translates that Aarifa is mocking my questions. I ask her to elaborate on what she means*,* pointing to the space around her*.* This time her voice rises a pitch and her eyebrows furrow. Now visibly frustrated she answers, “This is what we do: we cook, we clean, we take care of the house, we take our children to school, we cook again, and we sleep.” (excerpt from field notes, November 23, 2023)

For Aarifa, a young Rohingya refugee mother in her 20s, her everyday life is dominated by the relentless demands of domestic tasks. Like her, many Rohingya refugee women I met undertake a complex daily routine of unpaid tasks in conditions of extreme scarcity. Tellingly, many of my interviews were cut short or requests denied because women had to complete household chores and had to look after their young children. Aarifa's statement highlights the repetitive nature of these essential practices, including cooking, cleaning, caregiving, and resource management, which she explicitly associates with her identity as a woman. These practices are crucial to maintaining a sense of stability and sustaining intergenerational reproduction, especially in severely resource-constrained environments (Parreñas 2012). However, her exasperated and mocking tone underscores the all-encompassing nature of this work and its simultaneous undervaluation both within her community and in societal discourse.

This was reaffirmed during my conversation with Afzal, a Rohingya refugee man in his 30s, and *zimmedar^5^* (community leader) of the camp:

In our community, especially among the Rohingya, most of the ladies are housewives. They take care of their households, cook food, and manage their homes in this way. (interview, October 28, 2023)

Afzal's statement reflects how gendered expectations around caregiving are naturalized, structuring the lives of Rohingya women. This also serves as a form of governance within the community, maintaining social order and cultural continuity amidst displacement. Oppressive power structures within Rohingya refugee camps, including those in Bangladesh, perpetuate traditional gender roles and values, exposing women to exploitation even within their communities (Akhter and Kusakabe 2014). The naturalization of women's unwaged care work reproduces a strict gendered division of labor that confines Rohingya women to the home and centers their identities around attending to their children.

This division of labor is frequently challenged by the financial, material, and legal insecurities that the Rohingya refugees face every day because of their statelessness in Delhi. For many women, the lack of basic facilities, such as housing, toilets, healthcare, and education severely affects their ability to mother and raise their children. As explained by Khalida Begum, a single mother of four children in her thirties:

[…] Look, despite sitting at home, so many people watch us, going past us. We are women, and women should always live with dignity. But we don't even have a bathroom […] We don't even have a proper house with multiple rooms. We have so many children, some of them are babies, and everyone sleeps together. Is this not a compulsion? It's not just about earning and eating well […] What about the future? What will be the life of our children? I fall sick thinking about this. (interview, October 23, 2023)

This statement reveals how systemic marginalization impacts women's experiences in the settlement, where administrative neglect and material insecurity compromise their capacities to fulfill their culturally expected roles as nurturers and protectors. Khalida Begum also describes an acute lack of privacy within these overcrowded spaces which she associates with a loss of dignity and increased insecurity. In this sense, Rohingya refugee women continuously negotiate the blurred boundaries between the public and private which shapes their everyday roles and identities. Moreover, displacement from Myanmar has eroded social networks, including relationships with relatives, friends, and their community, that are crucial for supporting women's care labor (Pearson and Kusakabe 2012). In a community fraught with tension over limited resources, women often face these demands in isolation.

Therefore, the layered insecurities reproduced by cultural norms and financial, material, and social insecurities in the aftermath of displacement have placed additional burdens on Rohingya women, forcing some of them to seek employment. Culturally, Rohingya women are expected not to engage in gainful employment outside the settlement due to an interplay of social, cultural, and religious ideologies. Even when meager incomes demand additional contributions, this restriction is enforced by husbands, mothers-in-law, and neighbors, as well as by women's apprehensions about safety in the city and language barriers. Indeed, there are deep-seated patriarchal norms against women who work or seek education outside the settlement, subjecting them to suspicions, taunts, and ire from the community. Thus, for many Rohingya women, mothering and raising families within the settlement involves making a continuous choice between the social and cultural expectation to stay at home and financial precarity, which forces them to seek employment.

For instance, for Taslima, a widowed 50-year-old mother who works as a janitor in a restaurant in New Delhi, this tension has created a profound sense of loss and frustration:

I don’t have a husband […] If we had a man earning in our house as well, I could just sit at home and do household chores. I don’t get to spend time with my daughter, the whole day goes to work. After coming home from work, there is barely any time to even cook. In households where there is a man, it is the man's job to work, bring food, or any other items. A woman's job is to cook at home, take care of their children, send them to school, and help them build a future. (Interview, October 31, 2023)

This excerpt reveals the contradictions that shape Rohingya women's lives in displacement. Taslima simultaneously resists gender roles by working outside the home, while also reinforcing them through her belief that domestic tasks are a “woman's job.” Her statement reveals how women both internalize and reproduce patriarchal norms. This excerpt also illustrates the contradictions between the effort and time needed for everyday social reproduction and care labor and the labor needed to navigate financial precarity by seeking waged work outside the settlements. For Taslima, this tension has resulted in a breakdown in traditional family structures within her community and affected her ability to mother according to cultural expectations.

However, this tension also provides spaces for challenging these traditional roles and pushing the boundaries of motherhood. For instance, Mahira, a 26-year-old married mother of four children, reflected on her double burden of mothering and working as a local vendor outside the settlement to supplement her husband's irregular income:

What I am doing is right, I am not engaging in theft or doing anything wrong. If someone is saying something behind my back, let them say it. What difference does it make? I don’t have any problem with it. I only want to educate my children through my income. (Interview, October 22, 2023)

Mahira's statement encapsulates the complexities of the insecurities faced by Rohingya refugee women, particularly mothers who defy traditional gender roles and norms to secure better futures for their children in displacement. Her statement exemplifies the contradiction between gendered expectations around motherhood and the demands of displacement, which is at odds with the various material, social, and emotional insecurities imposed by precarious living conditions. It illustrates her subtle defiance of these expectations, driven by her sense of responsibility as a mother to secure her children's futures. Indeed, research has illustrated that motherhood reorients migrant women towards the future (Pearson and Kusakabe 2012), and that framing one's actions as maternal concerns may provide a space for the exercise of agency to women (Prasch 2015). Yet, this internalized expectation of motherhood also reinforces the notion that the burden of care and survival rests on women, thereby reproducing the very insecurities Mahira seeks to negotiate.

Thus, Rohingya women continuously negotiate various gendered expectations around motherhood, which are shaped by intersecting class, cultural, and familial norms. Although conforming to these expectations legitimizes Rohingya women's roles within the community, they simultaneously govern their everyday practices, their mobility, and their identities. In displacement, these expectations intensify the insecurities experienced by Rohingya women. Indeed, Rohingya women's responses to these challenges are varied and illustrate the tenuous character of mothering in displacement.

### Negotiating Security Amidst Insecurity

In Delhi, the roles and identities of Rohingya women as mothers are shaped not only by the intersecting gendered expectations within their community but also by the disruptive and exclusionary practices of the Indian State, such as threats of detention and deportation and routine visits by the police and immigration officials. Not only are the refugee settlements in Delhi spaces of routine surveillance, but they are also spaces of state abandonment and neglect. Here, basic infrastructure, such as housing, toilets, and emergency response to crises (such as floods and fires) remain inadequate. Such neglect is often a form of deliberate structural violence that specifically targets the means of survival of particular racial, ethnic, or cultural minorities to disempower them and create hostile environments (Davies, Isakjee and Dhesi 2017).

A notable concern among Rohingya refugees in Delhi is the provision of housing infrastructure. Since 2017, major Rohingya settlements in the city have been ravaged by fires — common in refugee settlements globally due to the high density of the settlements, the flammability of the materials used to construct homes, and tensions within the community (Doyel 2021). In Delhi, regular instances of fires have compelled Rohingya families to remain in a state of perpetual displacement and readiness for future evictions. During my time in these settlements, the area available to the Rohingya for constructing their homes had decreased due to fires and evictions, creating congested settlements — at times over 50 families living in a small patch of land. The precariousness of housing is a particular concern for Rohingya women whose roles, identities, and mobility are entangled with the materiality and spaces of the home, as mentioned in the previous section.

One such instance was narrated to me by Shahida, a 30-year-old Rohingya mother of two, who lost all her belongings in the last instance of fires in the settlement in 2023:

In January someone set the door on fire in my house. At 10 PM, everyone had gone into their shanties and locked the doors. At around 1:00–1:30 AM [...] I am not sure who did it. God only knows. I was sleeping upstairs in my two-storey wooden house. We don’t know who sets these fires, whether they are from inside or outside the community. This keeps bothering us. We don’t sleep at night; we stay up until two or three in the morning afraid of the fire and guarding our homes. I am a single parent, I have children at home, and if something happens suddenly, who will save our life? […] Even when we are not on guarding duties, we stay awake at night. When there's a fire, we leave everything behind; we just grab the children and run. (Interview, October 17, 2023)

Shahida's narrative encapsulates the expansion of Rohingya mothers’ roles beyond caregiving to encompass safeguarding her family from imminent physical threats, such as fires. Her practice of “staying awake” and “guarding her home at night” illustrates how Rohingya women often take on additional security roles. Despite nightly police patrols in the settlements, as reported by some of my participants, Shahida's persistent concerns for maintaining the safety of her family underscores the pervasiveness of the insecurity within the settlement despite routine surveillance. It also highlights a distrust of state services because of a failure of the police to stop regular incidences of fires.

While Rohingya refugee men also engage in such practices, women experience security/insecurity in a deeply personal way and as uniquely tied to their maternal identities. For instance, one participant confessed her feelings of “failing as a mother” because of her inability to protect her children from this constant state of insecurity. On this subject, Shahida explained to me that the first thing she did when her house caught fire was to secure the birth certificates of her children. Smiling widely as she recounted that part of the story, she explained the importance of these documents for her stateless children. As their only proof of identity, birth certificates ensure that Shahida's family is eligible for UNHCR's resettlement program. When I asked Shahida if the other women did the same, she responded by saying: “I am smart and think of my children's future, the other women did not” (Excerpt from field notes, October 10, 2024). Previous research has illustrated the significance of paperwork and documentation to the bureaucratic governance of migration and for creating hierarchies of inclusion and exclusion against refugees (Andreetta and Borrelli 2024). Thus, in displacement, the expectations around being a “good mother” among Rohingya women expand to incorporate insecurities that result from the lack of documentation and the uncertainties about future resettlement.

In addition to negotiating material and physical insecurities, Rohingya women must also navigate the emotional and psychological insecurities that arise from their statelessness. Thus, for Mumtaz Begum, a widowed 30-year-old mother of four, an essential part of her everyday life was helping her children make sense of and adjust to the realities of their displacement. She explains:

Our children ask us: Where is our home? Where is our village? What does ‘Rohingya' mean? We don’t want to disappoint our children, so we hide the reality of their past from them. If we tell them about the violence and injustice we have faced and continue to face daily, the child will not be able to focus on their studies…it may scar them mentally and emotionally. We tell them that we live here because we are poor. We tell than if they study, they will get a job, build their own house, and live a good life. (Interview, October 17, 2023)

This statement highlights the crucial role mothers play in helping their families come to terms with the realities of displacement (Al-Dabbagh 2022). Like Mumtaz Begum, several of the women interviewed, preferred to remain silent about their violent past, instead choosing to reorient themselves and their children to the future. Simultaneously, the association of the precarious conditions of living with poverty also points towards an internalization of everyday insecurities in Delhi. Such strategies can be choice, a form of agency or resistance, against the exclusionary practices of states and other institutions, or simply strategies to cope with the trauma of prolonged violence and insecurity (Blomqvist, Olivius and Hedström 2021).

Everyday motherhood not only includes managing emotional trauma but also shaping children's sense of belonging. Mumtaz Begum, for instance, identified herself as “*Burma-wala*” (Burmese) because she was born in Myanmar, yet she considered her children Indian because as they were born in India. By framing their children's identities around place of birth rather than ancestry or legal citizenship, mothers like Mumtaz construct alternative narratives of inclusion amidst exclusionary discourses that label Rohingya refugees as ‘illegal intruders' in India. Mumtaz's statements exemplify how refugee mothers become agents of subtle identity formation in response to nationalist and securitized discourses that exclude them from both Myanmar and India.

The narratives in this section illustrate how the Indian State is simultaneously present through routine surveillance and exclusionary practices yet absent in providing a meaningful sense of safety or resources to refugees. As a result, refugees — particularly women — are forced to navigate security and insecurity on their own. While both Rohingya refugee men and women take on these additional burdens, for Rohingya women security is not just about physical safety, but is tied to their responsibilities as mothers and is an extension of their caregiving roles. Thus, even within the framework of formal refugee governance, women's mothering roles and practices are mobilized to sustain the survival of refugee communities against the exclusionary policies of states. Rather than the stereotypical notion that mothers are inherently caring or loving, these mothering practices are a confluence of layered material, class and gender-based, and non-citizenship insecurities that marginalize Rohingya women in India.

### Mobilizing Motherhood Within Humanitarian Governance

The everyday experiences of Rohingya women in Delhi are also shaped by their regular interactions with non-state actors. Settlements are frequented by various humanitarian organizations, like the UNHCR and its partners, which implement development and aid delivery initiatives for supporting refugee communities. Previous research has revealed that representing refugee women as vulnerable mothers and caregivers is often a strategic tool to mobilize international support behind humanitarian interventions and is crucial to neoliberal frameworks of refugee governance (Johnson 2011). Highlighting refugee women's vulnerabilities depoliticizes securitized refugees while also constructing them as feminized figures in need of humanitarian aid (Johnson 2011). Moreover, the Indian government's non-recognition of Rohingya refugees complicates the involvement of humanitarian actors within the settlements who are wary of state scrutiny. Thus, mobilizing Rohingya women's identities as mothers can often be a way to access communities without appearing to challenge political narratives. During my fieldwork, NGO officials often advised me to foreground the focus on mothers and motherhood in my research to reduce the likelihood of state scrutiny and prevent arousing the suspicion of the local police.

However, far from being apolitical, the mobilization of motherhood for humanitarian purposes is entrenched in the gendered governance of refugees. Several interventions in Rohingya settlements in Delhi are explicitly gender-sensitive programs, targeting women and children, offering education, awareness of reproductive health, vocational training, and psychosocial support. While these interventions can provide critical resources and spaces for Rohingya women to connect and cope with their increasing insecurities, they simultaneously reproduce gendered expectations around motherhood that may not align with Rohingya women's everyday realities.

For instance, during visits to an NGO center in a low-income neighborhood in Delhi, I observed how ideas around motherhood, and related issues of reproduction, are subtly reproduced through a focus on women's maternal identities and bodies within health camps:

Today, Dr A [a female gynaecologist] is explaining the basics of reproductive health to a group of migrant and refugee women from the neighbourhood. A few Rohingya women have managed to attend, sitting quietly in the back […] I notice that there are no men in the room. Dr. A drones on monotonously and occasionally points to a painting of the female reproductive system stuck to the pin board behind her and talks about the benefits of various contraceptives, the essentials to ensuring healthy pregnancies, and the importance of focusing on the well-being of their children and themselves. Dr. A is struggling to translate medical words from English to Hindi and frantically looks to the other volunteers for help to translate her explanations […] In the end, she hands everyone a colourful A5-size sheet of paper with a list of fruits, vegetables and nutrients women should include in their diet. (Excerpt from field notes, May 16, 2023)

By specifically targeting women in campaigns around reproductive health, family planning, and childcare, humanitarian interventions can reinforce women's roles as primary caregivers. While disseminating critical information, the regular absence of men within these sessions highlights how humanitarian actors reproduce strict gender roles within these communities. Similarly, some of the educational interventions I attended — which sought to help refugee women achieve a basic level of literacy — were often promoted as measures to “strengthen families by educating mothers.” Such initiatives link women's everyday roles as mothers to broader narratives of insecurity and displacement, while implicitly placing the burden of survival and resilience on women.

These examples also illustrate the gap between humanitarian actors’ and refugee women's understandings of their priorities. This is demonstrated not only by the significant language barriers that often exist between refugees and NGO officials as in the case of the health camp quoted above, but also in the dissonance between refugees’ financial precarity and the advice of the medical professional to incorporate certain unaffordable food items in their diet. Even as humanitarian organizations and non-state actors provide support, they can neglect the structural and material insecurities that shape Rohingya women's lives and create reductive and racialized stereotypes around motherhood. For instance, during a conversation with a female legal professional who often works with Rohingya women, she acknowledged the immense hardship women face while also saying that: “I don’t think they [Rohingya mothers] are raising their children at all […] They are like children themselves” (Interview, October 28, 2024). The infantilization of refugees in this manner and judgments on their maternal competence further marginalize refugee women and reinforce power hierarchies between them and non-state actors.

Some Rohingya women alluded to a sense of fatigue over these humanitarian interventions which failed to address material concerns while also challenging the community's conservative religious beliefs against contraception. Despite this, Rohingya women continued to attend these sessions in the expectation of food, cosmetics, and other aid. Crucially, this reveals how humanitarian organizations produce specific gendered expectations, while also shaping and regulating women's access to resources in the refugee settlement through aid delivery (Saltsman 2023). Indeed, these additional burdens can complicate the everyday insecurities among women who not only negotiate gendered expectations at the community level but also the regulatory and idealized standards set by humanitarian actors.

Regular interactions with humanitarian organizations in Delhi have also created new opportunities for Rohingya women. Several NGOs recruit refugee volunteers for administrative work. This includes recruiting refugees to conduct monthly community meetings to raise awareness on issues, such as domestic violence, teenage marriages, and generating certain kinds of knowledge for humanitarian agencies by registering refugees, conducting family surveys, interviewing families, and providing interpretation services. In Delhi, NGOs often rely on women volunteers for these sessions. Typically, these recruitments are un(der) paid and can be exploitative, offloading the tasks central to refugee governance onto refugees to promote self-reliance (Easton-Calabria and Omata 2018).

Mumtaz Begum, who was recruited as a community leader, revealed the crucial role she plays in supporting NGOs by performing a wide range of tasks: Talking about her responsibilities, she adds:

[…] If there is an emergency at night, if someone falls ills, or if a woman is in labor, if anyone in the community needs an ambulance, they come to me. Everyone comes to me whenever they have any problem. […] I think this is why I receive support from the NGOs. […] I don’t care about the money. I see my community as my family. They have given me so much respect by making me the leader. (Interview, October 17, 2023)

As this excerpt reveals, organizations working within the Rohingya settlements have gradually incorporated women like Mumtaz Begum into the folds of humanitarian governance. Tasks such as resource management, taking care of community members when they are in distress, mediating conflicts, and managing households mirror the unpaid and undervalued caregiving and social reproduction tasks she normally performs at home. Thus, her employment as a community leader has expanded her caregiving responsibilities beyond her family, extending her maternal role to the entire community. Her description of the community as a “family” and her sense of “care(ing)” for the community instead of financial remuneration for her work, exemplify how this labor is framed as a natural extension of women's unpaid and undervalued caregiving responsibilities within the context of humanitarian governance. On the one hand, volunteering has provided women with opportunities to forge social networks, advocate for their rights, and garner the “respect” of the community. However, Mumtaz Begum's reflection that “this why she receives support from the NGOs” exemplifies how humanitarian actors entrench women's dependency while normalizing the exploitation of their un(der) paid labor.

Hamida, a 24-year-old married mother of three children and a community volunteer who accompanies Rohingya women to the hospital as a translator explains how her recruitment impacts her time with her children:

I have to go to work every day. Most days I come back only at 8 PM, even on the weekends. I must be always prepared to accompany pregnant women or children in the settlement to the hospital, stay with them, and translate everything to the doctor. It takes up all my time. I rarely spend time with my children, except a few hours on the weekends. (Interview, Hamida, November 18, 2023)

Hamida's new role within the settlement involves choosing between caring for her community and her responsibilities at home as a mother. This extension of women's roles also leaves many women debilitated and depleted (Rai, Hoskyns and Thomas 2013), affecting their ability to perform the routine practices of motherhood as Hamida explains through her lamentation of being unable to “spend time” with her children. The narratives in the section further illustrate the broader trends within the neoliberal governance of refugees which appropriates the labor of refugees by offloading responsibilities of surveillance, monitoring, and crisis response onto the communities themselves (Tazzioli 2024). Therefore, humanitarian interventions can be both a form of support and a mode of control and exploitation. Significantly, motherhood emerges as an important site for the governance of refugees where women continuously negotiate the various gendered expectations reproduced by non-state actors (Clark 2016).

## Conclusion

Prolonged displacement and statelessness intensify the gendered insecurities experienced by Rohingya refugee women raising families on the margins of nation-states. The narratives in this paper illustrate that motherhood serves as a crucial site for the governance of refugee women. Analyzing these experiences through the lens of motherhood reveals how intersecting factors, such as class, cultural and familial norms, state securitization, and non-state interventions, shape Rohingya refugee women's roles, identities, and practices as mothers within refugee settlements and camps. These factors not only expose women to new gendered insecurities because of their statelessness but also reproduce existing ones, reinforcing gendered expectations and creating conditions that exploit and undervalue their everyday care and social reproduction labor.

My findings demonstrate the pervasive relationship between motherhood, gendered insecurities, and the governance of refugees. Firstly, the contradictions between the traditional and gendered expectations around motherhood and the lived realities of prolonged displacement regulate Rohingya women's everyday practices, restricting their access to essential resources and mobility while placing additional financial, material, and emotional burdens on them. Secondly, amid increasing insecurities in Delhi, Rohingya women's caregiving labor is mobilized to fill gaps resulting from state abandonment, even as their securitized and racialized maternal bodies are excluded from the nation-state. Finally, motherhood is also a site of humanitarian interventions, where non-state actors play a pivotal role in governing Rohingya women through various development programmes. These processes can be violent, further entrenching gender hierarchies in ways that exploit and drain women's limited resources.

While many Rohingya women reify these gender roles, motherhood also becomes a site of agency, creating space for resisting patriarchal stereotypes, gendered restrictions, and marginalization. Ayhan and Colpitts-Elliott (2024) document activism among Rohingya women in Bangladesh who resist neoliberal governance by cultivating emotional resilience, facilitating knowledge sharing, and fostering networks of mutual care and protection. While this has not been the case in India, my research explores whether similar forms of agency and resistance might emerge, offering nuanced insights into the gendered dimensions of displacement and refugee governance in diverse contexts. Therefore, paying closer attention to the various dimensions of motherhood can reveal the deeply gendered nature of irregular migration and governance in countries in South Asia as thousands of women continue to mother from the margins.

## Notes

1 Research participants have been given pseudonyms for anonymity.

2 A slum dwelling made of tarpaulin, wood, iron, and other materials.

3 This research was approved by the ethics committee at the institution where the author is based. All interviews were conducted with the informed verbal consent of participants.

4 These ages are not exact. Many women revealed that they were unsure about their age because they did not have birth certificates.

5 In Hindi and Urdu, the word *zimmedar* means “responsible.” In the Rohingya community in Delhi, it refers to their community leader who is responsible for maintaining harmony, mediating disputes, liaising with the UNHCR and state authorities, and distributing aid.
